# Outer layer of Vb neurons in medial entorhinal cortex project to hippocampal dentate gyrus in mice

**DOI:** 10.1186/s13041-024-01079-5

**Published:** 2024-02-05

**Authors:** Naoki Yamamoto, Jun Yokose, Kritika Ramesh, Takashi Kitamura, Sachie K. Ogawa

**Affiliations:** 1 Department of Psychiatry, University of Texas Southwestern Medical Center, Dallas, TX 75390 USA; 2 Department of Neuroscience, University of Texas Southwestern Medical Center, Dallas, TX 75390 USA; 3 Peter O’Donnell Jr. Brain Institute, University of Texas Southwestern Medical Center, Dallas, TX 75390 USA

**Keywords:** Medial entorhinal cortex, Hippocampus, Anterior thalamus, Learning, Memory, Ctip2, PCP4, Retrograde tracer

## Abstract

Entorhinal cortical (EC)-hippocampal (HPC) circuits are crucial for learning and memory. Although it was traditionally believed that superficial layers (II/III) of the EC mainly project to the HPC and deep layers (V/VI) receive input from the HPC, recent studies have highlighted the significant projections from layers Va and VI of the EC into the HPC. However, it still remains unknown whether Vb neurons in the EC provide projections to the hippocampus. In this study, using a molecular marker for Vb and retrograde tracers, we identified that the outer layer of Vb neurons in the medial EC (MEC) directly project to both dorsal and ventral hippocampal dentate gyrus (DG), with a significant preference for the ventral DG. In contrast to the distribution of DG-projecting Vb cells, anterior thalamus-projecting Vb cells are distributed through the outer to the inner layer of Vb. Furthermore, dual tracer injections revealed that DG-projecting Vb cells and anterior thalamus-projecting Vb cells are distinct populations. These results suggest that the roles of MEC Vb neurons are not merely limited to the formation of EC-HPC loop circuits, but rather contribute to multiple neural processes for learning and memory.

**Supplementary Information:**

The online version contains supplementary material available at 10.1186/s13041-024-01079-5.

## Main

In humans and rodents, the entorhinal cortical (EC)-hippocampal (HPC) networks are crucial for the formation and recall of episodic memory [1]. Identification of cell-type specific projections in the EC-HPC networks is critical for understanding the neural process and the computations underlying learning and memory. The hippocampus can be divided into the dentate gyrus (DG), CA3, CA2, and CA1 regions [2, 3]. It has been considered that the superficial layers (II/III) of EC mainly project to the HPC, while the deep layers (V/VI) receive the input from the HPC to provide telencephalic projections [4]. For example, Reelin^+^ cells in layer II of the EC project to the hippocampal DG, CA3, and CA2 [4–6]. Pyramidal cells in layer III of the EC directly project to the hippocampal CA1 [4, 7]. A subpopulation of Wolfram syndrome 1 (Wfs1) / CalbindinD-28 K (CalB)^+^ pyramidal cells in layer II of the EC project to the inhibitory neurons in the hippocampal CA1 area [4, 8, 9]. On the other hand, in contrast to the superficial layers, the cell-type specific projection pattern for the deep layers of the EC are beginning to be studied. Layer V can be separated into two sublayers: Va and Vb [4, 9, 10]. Vb neurons are known to function as a local projection to superficial layers in the EC [9, 11, 12], and Va neurons project to telencephalic structures [4, 9–11]. Previous studies raised the possibility that some neurons in the deep layers of the EC also may project back to the HPC [13, 14]. Utilizing molecular markers for deep layers and viral-based neural tracing, recent studies revealed that Va neurons collaterally project to telencephalic structures as well as the hippocampal CA1 area [15]. Furthermore, VI neurons project to the hippocampal CA1, CA2, CA3, and DG areas [16]. However, it remains unknown whether Vb neurons in the EC also project to the HPC. In this study, we investigated whether Vb neurons in the medial EC (MEC) project to the HPC.

**Fig. 1:**
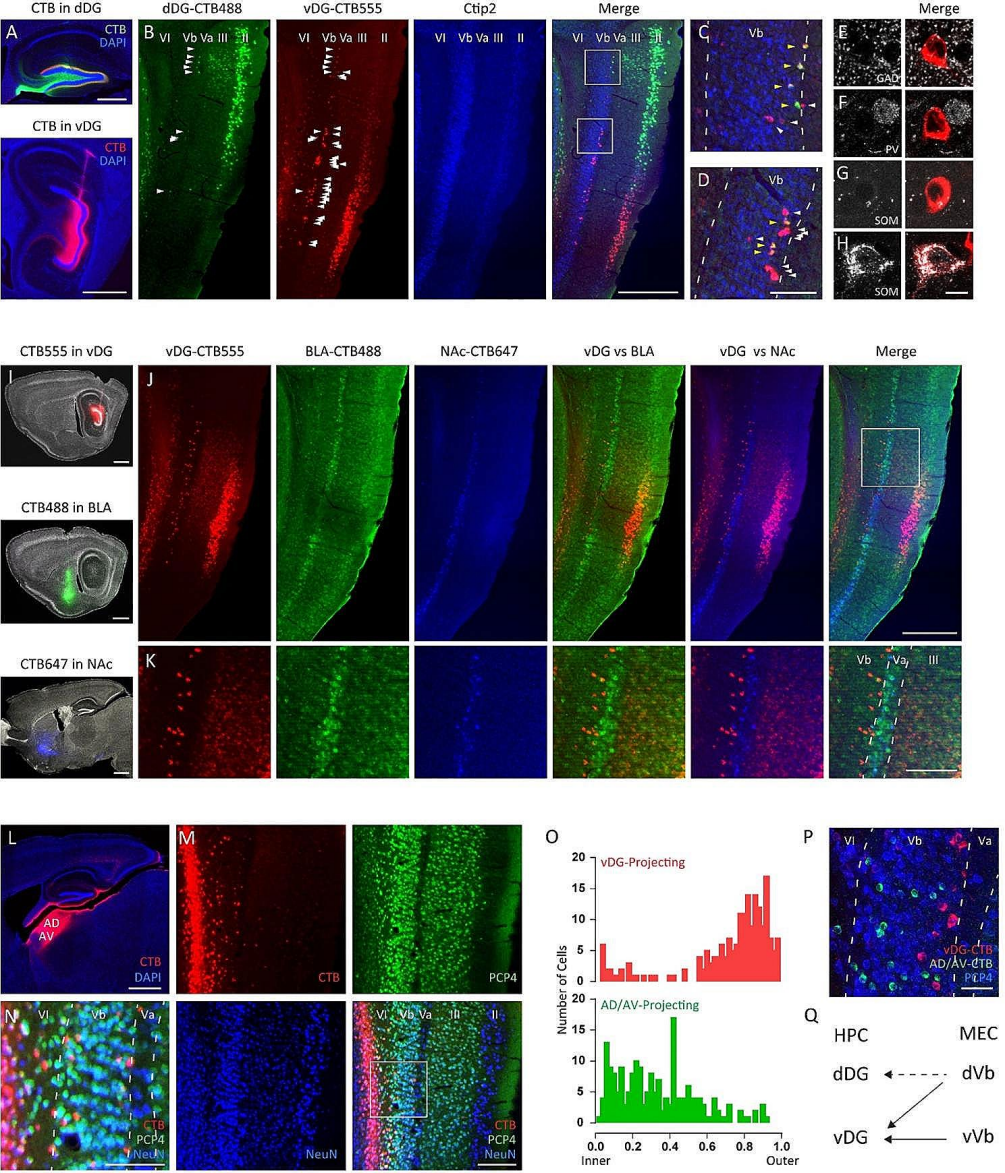
Outer layer of Vb neurons in medial entorhinal cortex project to hippocampal dentate gyrus in mice. (**A**) Injection of CTB488 and CTB555 into dorsal (top) and ventral DG (bottom), respectively. CTB488 (green). CTB555 (red). DAPI (blue). Scale bar, 500 μm (top), 1 mm (bottom). (**B**) Parasagittal sections of the MEC labeled with CTB488 (green), CTB555 (red) and immunostained with Ctip2 (blue). Arrowheads indicate CTB488^+^ cells and CTB555^+^cells in Vb, individually. Scale bar, 500 μm. (**C–D**) Magnified images of B, top square (**C**) and bottom square (**D**), respectively. Yellow arrowheads indicate CTB488 and CTB555 double positive cells. White arrowheads indicate CTB555 single positive cells. Scale bar, 100 μm. (**E–H**) Immunohistochemistry for CTB555^+^ cells using GABAergic interneuron makers, respectively (left). Merged images of left panel and CTB555^+^ cell, individually (right). (**E**) GAD67, (**F**) Parvalbumin, (**G**–**H**) Somatostatin. Example image of somatostatin negative CTB555^+^ cell (**G**) and somatostatin positive CTB555^+^ cell (**H**). Scale bar, 10 μm. (**I**) Injection of CTB555 (red) into vDG (top), CTB488 (green) into BLA (middle), and CTB647 (blue) into NAc (bottom), respectively. Scale bar, 1 mm. (**J**) Parasagittal sections of the MEC labeled with CTB488, CTB555, CTB647. Scale bar, 500 μm. (**K**) Magnified images of J, respectively. Scale bar, 200 μm. (**L**) Injection of CTB555 into AD/AV. Scale bar, 1 mm. (**M**) Parasagittal sections of the MEC labeled with CTB555 (top left, red) and immunostained with PCP4 (top right, green), NeuN (bottom left, blue) and merged image (bottom right). Scale bar, 200 μm. (**N**) Magnified image of square area in M. Scale bar, 100 μm. (**O**) Distribution of vDG-projecting CTB^+^ cells (top, *n* = 7 mice) and AD/AV-projecting CTB^+^ cells (bottom, *n* = 6 mice) in MEC Vb which represented by 50 bins through the outer to the inner layer of Vb. (**P**) Parasagittal section of the MEC Vb labeled with CTB488 (green) and CTB555 (red) and immunestained with PCP4 (blue), which CTB488 and CTB555 were injected into AD/AV and vDG, respectively. Scale bar, 50 μm. (**Q**) Summary for projection of MEC layer Vb neurons into hippocampus

We injected retrograde tracers, Cholera Toxin Subunit B (CTB) 488 and CTB555, into the dorsal (AP: -2.00, ML: 1.30, DV: -2.00) and ventral DG (AP: -3.70, ML: 2.90, DV: -3.50) (Fig. 1A) of 6–10 weeks old C57BL6J male and female mice (JAX:000664), respectively (dDG: 100nl, vDG: 70nl, 0.5% wt/vol, Invitrogen). Four to six days after the injection, these mice were deeply anesthetized with a cocktail of ketamine (75 mg/kg)/dexmedetomidine (1 mg/kg) and then transcardially perfused with 4% paraformaldehyde (PFA) in PBS. Brains were extracted and post-fixed overnight in 4% PFA in PBS at 4 °C and then sectioned at a thickness of 60 μm using a vibratome (Leica). We found both CTB488^+^ and CTB555^+^ cells in layer II of MEC (MECII); CTB488^+^ cells were observed in the dorsal part of MECII while CTB555^+^ cells were found in the ventral part of MECII (Fig. 1B), as previously demonstrated [2, 3, 5], indicating that we successfully injected these tracers into the dorsal and ventral DG. We also found small population of CTB488^+^ and CTB555^+^ cells in the layer V of MEC, while CTB555^+^ cells were more abundant than the CTB488^+^ cells (Fig. 1B). There were a partial overlap between CTB488^+^ and CTB555^+^ cells; 27.03% of CTB488^+^ cells were labeled with CTB555 while 5.99% of CTB555^+^ cells were labeled with CTB488 (Fig. 1B–D, total 37 CTB488^+^ cells and 167 CTB555^+^ cells, 5 mice). We also found that CTB488^+^ cells were preferentially located in the dorsal part of MEC, while CTB555^+^ cells were distributed through the dorso-ventral axis (Fig. 1B, CTB488^+^ cells; dorsal 50.00%, intermediate 36.36%, and ventral 13.64%, total 22 cells from 4 mice, CTB555^+^ cells; dorsal 22.92%, intermediate 46.88%, and ventral 30.21%, total 96 cells from 4 mice). These results indicate that layer V of MEC neurons project to dorsal and ventral hippocampal DG.

To identify whether Vb neurons in the MEC project to DG, we next examined the immunohistochemistry for Ctip2, a marker for MEC Vb neurons [9] (rat anti-Ctip2 antibody; ab18465, abcam, 1/300 dilution) and found that both CTB488^+^ and CTB555^+^ cells were colocalized with Ctip2 (Fig. 1B–D). 0.56 ± 0.14% of neurons were CTB488^+^ and 2.48 ± 0.32% of neurons were CTB555^+^ in MEC Vb (*N* = 4). Importantly, the outer layer of Vb neurons was preferentially labeled with CTBs (Fig. 1C–D). These results indicate that neurons in the outer layer of Vb preferentially project to both dorsal and ventral DG (vDG), with a significant preference for the vDG. To identify the cell-types of DG-projecting Vb cells, we examined immunohistochemistry using inhibitory neuron makers; GAD67, parvalbumin and somatostatin (Fig. 1E–H) **(**mouse anti-GAD67 antibody; MAB5406, Millipore, 1/200 dilution, mouse anti-parvalbumin antibody; PV235, SWANT, 1/200 dilution, rat anti-somatostatin antibody; MAB354, Millipore, 1/200 dilution). There were no DG-projecting cells which colocalized with GAD67 (Fig. 1E, dDG-projecting cells; 0%, 0 out of 29 cells from 7 mice, vDG-projecting cells; 0%, 0 out of 364 cells from 7 mice) or parvalbumin (Fig. 1F dDG-projecting cells; 0%, 0 out of 21 cells from 6 mice, vDG-projecting cells; 0%, 0 out of 369 cells from 6 mice). Only 3 vDG-projecting cells were colocalized with somatostatin (Fig. 1G, H, dDG-projecting cells; 0%, 0 out of 31 cells from 7 mice, vDG-projecting cells; 0.70%, 3 out of 429 cells from 7 mice). These results suggest that the majority of DG-projecting Vb neurons in the MEC are excitatory neurons.

Va neurons project to the basolateral amygdala (BLA) and nucleus accumbens (NAc) [4, 8, 9]. To examine the layer specificity in the MEC, we injected CTB488, CTB555, and CTB647 into the BLA (AP: -1.40, ML: 3.40, DV: -5.00), vDG (AP: -3.70, ML: 2.90, DV: -3.50), and NAc (AP: 1.15, ML: 0.70, DV: -4.70) of 6–10 weeks old C57BL6J male mice (*N* = 4), respectively (BLA 100nl, vDG: 70nl, NAc: 300nl, 0.5% wt/vol, Invitrogen) (Fig. 1I). We found that vDG-projecting cells were never colocalized with BLA- or NAc-projecting cells (Fig. 1J, K), suggesting that Va neurons do not project to the vDG.

A previous study [9] showed that a subpopulation of Vb neurons projects to the anterior thalamic nuclei in mice. Therefore, to identify if DG-projecting Vb cells belong to the same population as anterior thalamus-projecting Vb cells, we injected CTB555 into the anterodorsal/anteroventral thalamic nucleus (AD/AV) (AP: -0.90, ML: 0.90, DV: -3.15) (150nl, 0.5% wt/vol, Invitrogen) of 6–10 weeks old C57BL6J male and female mice (*N* = 8) (Fig. 1L) and examined the immunohistochemistry for Purkinje cell protein 4 (PCP4), a marker for ECIII and ECVb [10], (rabbit anti- PCP4, Sigma, HPA005792, 1/300) and NeuN (chick anti-NeuN, abcam, ab134014, 1/1000) (Fig. 1M, N). In contrast to the DG-projecting Vb cells, which are preferentially located in the outer layer of Vb (Fig. 1O-P), anterior thalamus-projecting Vb cells were distributed from the inner to outer layer of Vb (Fig. 1O-P). Furthermore, double CTB injections into same mice (CTB488 in AD/AV and CTB555 in vDG, respectively, male and female 5 mice) demonstrated that there is no overlap between vDG-projecting cells and AD/AV-projecting cells in Vb (Fig. 1P, **0**% overlap, examined total 171 vDG-projecting cells and 213 AD/AV-projecting cells from 5 mice). These results suggest that DG-projecting Vb cells are different population from anterior thalamus-projecting Vb cells.

Vb neurons have been known to function as local projections to superficial layers in the EC to form the EC-HPC loop circuit [4, 9, 11, 12]. However, in this study, we identified that the outer layer of MEC Vb neurons directly project to both the dorsal and ventral DG, with a significant preference for the ventral DG (Fig. 1Q). DG-projecting Vb cells and anterior thalamus-projecting Vb cells are distinct populations and they have differential distribution patterns in Vb (Fig. 1O). Both male and female mice showed similar anatomical distribution. Although it has long been considered that only superficial layers of EC neurons project to the HPC, accumulating evidence [15, 16] including this study indicates that the subpopulation of neurons from all layers in the EC differentially provide significant projections to the HPC. Further studies will be required to understand the neural processes and the computations underlying learning and memory, based on the updated anatomical maps in the EC-HPC networks.

### Electronic supplementary material

Below is the link to the electronic supplementary material.

Supplementary Material 1

## Data Availability

Data for analysis will be made available by the corresponding author upon reasonable request.

## Abbreviations

AAV: Adeno-associated virus
AD: Anterodorsal thalamic nucleus
AV: Anteroventral thalamic nucleus
BLA: Basolateral amygdala
CalB: CalbindinD-28 K
CTB: Cholera Toxin Subunit B
Ctip2: Chicken ovalbumin upstream promoter transcription factor (COUP-TF) interacting protein 2
DAPI: 4′,6-diamidino-2-phenylindole
Ddg: dorsal Dentate gyrus
DG: Dentate gyrus
EC: Entorhinal cortex
GAD: Glutamate decarboxylase
HPC: Hippocampus
MEC: Medial entorhinal cortex
NAc: Nucleus accumbens
PCP4: Purkinje cell protein 4
PV: Parvalbumin
SOM: Somatostatin
vDG: ventral Dentate gyrus
Wfs1: Wolfram syndrome 1

## References

[1] Eichenbaum H (2000). A cortical-hippocampal system for declarative memory. Nat Rev Neurosci.

[2] Amaral DG, Witter MP. The three-dimensional organization of the hippocampal formation: a review of anatomical data. Neuroscience. 1989;31(3):571– 91. Epub 1989/01/01. 10.1016/0306-4522(89)90424-7. PubMed PMID: 2687721. 10.1016/0306-4522(89)90424-7 2687721

[3] Witter MP, Wouterlood FG, Naber PA, Van Haeften T (2000). Anatomical organization of the parahippocampal-hippocampal network. Ann N Y Acad Sci.

[4] Osanai H, Nair IR, Kitamura T (2023). Dissecting cell-type-specific pathways in medial entorhinal cortical-hippocampal network for episodic memory. J Neurochem.

[5] Tamamaki N, Nojyo Y (1993). Projection of the entorhinal layer II neurons in the rat as revealed by intracellular pressure-injection of neurobiotin. Hippocampus.

[6] Varga C, Lee SY, Soltesz I (2010). Target-selective GABAergic control of entorhinal cortex output. Nat Neurosci.

[7] Suh J, Rivest AJ, Nakashiba T, Tominaga T, Tonegawa S (2011). Entorhinal cortex layer III input to the hippocampus is crucial for temporal association memory. Science.

[8] Kitamura T, Pignatelli M, Suh J, Kohara K, Yoshiki A, Abe K, Tonegawa S (2014). Island cells control temporal association memory. Science.

[9] Surmeli G, Marcu DC, McClure C, Garden DL, Pastoll H, Nolan MF (2015). Molecularly defined circuitry reveals input-output segregation in deep layers of the Medial Entorhinal Cortex. Neuron.

[10] Kitamura T, Ogawa SK, Roy DS, Okuyama T, Morrissey MD, Smith LM, Redondo RL, Tonegawa S (2017). Engrams and circuits crucial for systems consolidation of a memory. Science.

[11] Ohara S, Onodera M, Simonsen OW, Yoshino R, Hioki H, Iijima T, Tsutsui KI, Witter MP (2018). Intrinsic projections of layer vb neurons to Layers Va, III, and II in the lateral and medial entorhinal cortex of the rat. Cell Rep.

[12] Iijima T, Witter MP, Ichikawa M, Tominaga T, Kajiwara R, Matsumoto G (1996). Entorhinal-hippocampal interactions revealed by real-time imaging. Science.

[13] Witter MP, Van Hoesen GW, Amaral DG (1989). Topographical organization of the entorhinal projection to the dentate gyrus of the monkey. J Neurosci.

[14] Deller T, Martinez A, Nitsch R, Frotscher M (1996). A novel entorhinal projection to the rat dentate gyrus: direct innervation of proximal dendrites and cell bodies of granule cells and GABAergic neurons. J Neurosci.

[15] Tsoi SY, Oncul M, Svahn E, Robertson M, Bogdanowicz Z, McClure C, Surmeli G. Telencephalic outputs from the medial entorhinal cortex are copied directly to the hippocampus. Elife. 2022;11. 10.7554/eLife.73162. PubMed PMID: 35188100; PMCID: PMC8940174. Epub 2022/02/22. 10.7554/eLife.73162 PMC8940174 35188100

[16] Ben-Simon Y, Kaefer K, Velicky P, Csicsvari J, Danzl JG, Jonas P (2022). A direct excitatory projection from entorhinal layer 6b neurons to the hippocampus contributes to spatial coding and memory. Nat Commun.

