# Supplementary material for: Outer layer of Vb neurons in medial entorhinal cortex project to hippocampal dentate gyrus in mice
Source: Mol Brain. 2024 Feb 5;17:5. doi: 10.1186/s13041-024-01079-5 (PMC10845563; doi:10.1186/s13041-024-01079-5)
Supplement: Supplementary file 1 — Supplementary Material 1 [file 13041_2024_1079_MOESM1_ESM.docx]

January 19, 2024

Dear Dr. Kaang,

We would like to submit our revised manuscript titled ‘Outer layer of Vb neurons in medial entorhinal cortex project to hippocampal dentate gyrus in mice’ by Yamamoto et al. for the micro report section of Molecular Brain.

We have performed all analysis and modification suggested by two reviewers and presented the results in both figures and text. We believe that this revised manuscript with additional analysis and descriptions are adequate for the publication of this study in Molecular Brain.

We would appreciate it very much your consideration for the publication of our manuscript and look forward to hearing from you.

Sincerely,

Sachie K. Ogawa
